# The regulatory function of the AAA4 ATPase domain of cytoplasmic dynein

**DOI:** 10.1038/s41467-020-19477-3

**Published:** 2020-11-23

**Authors:** Xinglei Liu, Lu Rao, Arne Gennerich

**Affiliations:** Department of Anatomy and Structural Biology and Gruss Lipper Biophotonics Center, Albert Einstein College of Medicine, Bronx, NY 10461 USA

**Keywords:** Biochemistry, Biophysics

## Abstract

Cytoplasmic dynein is the primary motor for microtubule minus-end-directed transport and is indispensable to eukaryotic cells. Although each motor domain of dynein contains three active AAA+ ATPases (AAA1, 3, and 4), only the functions of AAA1 and 3 are known. Here, we use single-molecule fluorescence and optical tweezers studies to elucidate the role of AAA4 in dynein’s mechanochemical cycle. We demonstrate that AAA4 controls the priming stroke of the motion-generating linker, which connects the dimerizing tail of the motor to the AAA+ ring. Before ATP binds to AAA4, dynein remains incapable of generating motion. However, when AAA4 is bound to ATP, the gating of AAA1 by AAA3 prevails and dynein motion can occur. Thus, AAA1, 3, and 4 work together to regulate dynein function. Our work elucidates an essential role for AAA4 in dynein’s stepping cycle and underscores the complexity and crosstalk among the motor’s multiple AAA+ domains.

## Abstract (web summary)

Cytoplasmic dynein contains three active AAA + ATPases (AAA1, 3 and 4), but only the functions of AAA1 and 3 are known. Here, we perform single-molecule fluorescence and optical tweezers studies and show that AAA4 controls the priming stroke of the motion-generating linker, which connects the dimerizing tail of the motor to the AAA + ring.

## Introduction

The cytoplasmic dynein motor complex (referred to here as dynein) is an intricate and ubiquitous biological nanomachine that is responsible for a vast array of functions including intracellular transport of organelles and signaling complexes, nuclear transport during neuronal migration, regulation of mitotic spindle length and positioning, and removal of checkpoint proteins from the kinetochore during cell division (reviewed in refs. ^1–4^). Thus, it is not surprising that dynein dysfunction has been linked to a growing number of human diseases termed “dyneinopathies”^5,6^ including malformation of cortical development (MCD)^7–9^, spinal muscular atrophy (SMA)^10,11^, SMA with lower extremity predominance (SMALED)^12,13^, and others^14–16^. However, dynein’s molecular mechanism, the regulatory functions of its subunits, and the molecular effects of disease mutations, remain largely unclear.

**Fig. 1:**
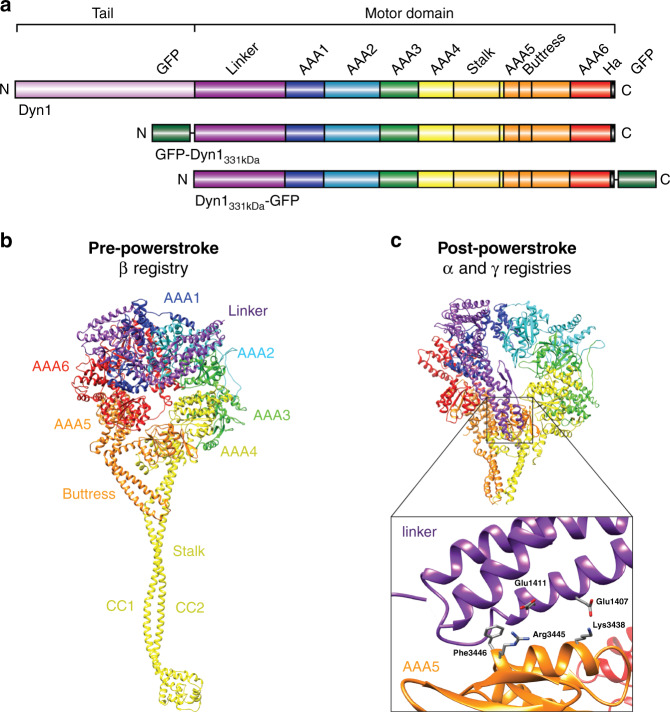
Cytoplasmic dynein domain organization and pre-powerstroke and post-powerstroke states in relation to the stalk-helix registrations of the dynein motor domain (MD). **a** Organization of the full-length cytoplasmic dynein heavy chain (HC) (a.a. 1–4092) and the tail-truncated monomeric constructs, GFP-Dyn1_331kDa_ and Dyn1_331kDa_-GFP (a.a. 1219–4092). **b** Dynein MD structure in the pre-powerstroke state (ADP.Vi, *Homo sapiens* cytoplasmic dynein-2; PDB entry 4RH7^60^). The linker is bent and close to AAA2 (left), and the stalk helices assume the weak microtubule (MT)-binding β registry as a result of the undocked linker^24^. **c** Post-powerstroke state (Apo, *S. cerevisiae* dynein; PDB entry 4W8F^62^). The linker is straight and docked on AAA5, and the stalk helices assume the strong MT-binding α registry or the γ registry with intermediate MT-binding strength. Interactions between hydrophobic linker residues (E1407 and E1411) and highly conserved AAA5 residues (F3446, R3445, and K3438) facilitate docking of the linker N-terminus on AAA5^62^ (PDB entry 4W8F^63^) (inset).

Dynein is the largest (~1.4 MDa) and most complex cytoskeletal motor protein. It is comprised of two identical heavy chains (HCs) and several subunits^4^. The dynein HC contains an N-terminal dimerizing tail domain and a C-terminal motor domain (MD) or “head” with six tandem-linked AAA+ ATPase modules (AAA: ATPase associated with various cellular activities) arranged in a ring (AAA1-6) (Fig. 1a). Only AAA1, 3, and 4 hydrolyze ATP^1^. Three elongated structures emerge from the AAA ring: A ~15-nm coiled-coil “stalk” that protrudes from AAA4 and separates dynein’s MT-binding domain (MTBD) from the AAA+ ring^17,18^, an antiparallel coiled-coil called the buttress^17^ (or strut^18^) that emerges from AAA5 and contacts the stalk, and a ~10-nm “linker” that extends from AAA1 and connects the AAA+ ring to the tail^19–21^. The linker undergoes conformational changes^19,22,23^ that generate unidirectional motion and force, and it also controls the buttress-mediated sliding of the stalk helices to shift between weak and strong MT-binding states^24^ (Fig. 1a, b).

Dynein can move processively (the ability to take multiple steps before dissociating^25^), and we are only beginning to understand how dynein generates continuous unidirectional motion under opposing forces. In mammalian dynein, activation by its largest cofactor, dynactin, together with a cargo adapter (e.g., BICD2) is required to convert dynein from a diffusive^26^/weakly processive^27^ motor to an ultraprocessive motor^28,29^. This activation involves realignment of the MDs into a parallel orientation where both MDs can readily bind the MT^30–33^. Once activated, processive movement can occur under load^34^, with one head binding the MT tightly while the other detaches and advances. Recent studies showed the cargo adapter-dependent recruitment of two dynein motors arranged side-by-side on a MT (i.e., four MDs in parallel)^35,36^. Recruitment of a second motor likely increases processivity by reducing the dissociation rate of the entire complex from the MT. While these studies provide important insights into the mechanisms that facilitate dynein processivity, a fundamental question remains: how do the convoluted interactions of dynein’s multiple AAA+ domains control the shifts between strong and weak MT-binding states of dynein’s MTBDs so that the force-bearing leading head stays bound to the MT while the partner head detaches and advances?

This question has been partially answered by studies that demonstrate that the stalk is a bidirectional communication pathway between the AAA+ ring and MTBD^24,37,38^. The nucleotide states of the MD affect MT binding and vice versa^39–41^. For example, it is known that ATP binding to AAA1 and ADP binding to AAA3 both reduce MT-binding strength^42^. Conversely, ADP binding to AAA1 has the opposite effect^42^. In addition, we^42^ and others^43^ reported that AAA3 acts as a “gate” for ATP-induced MT release. ATP binding to AAA1 induces MT release only if AAA3 is in the post-hydrolysis state. When force is applied to the linker, ATP binding to AAA3 “opens” the gate and allows AAA1-mediated, ATP-induced MT release^42^.

The stalk mediates this communication between the MD and the MTBD through shifts in the positions of the stalk helices relative to one another: coiled-coil 1 (CC1) and coiled-coil 2 (CC2) (Fig. 1b) slide against each other (up to 4.9 Å), assuming different stalk registries, which correspond to different MT-binding strengths^24,37,38,44^. The stalk-helix registries have been shown to influence both dynein-MT-binding affinity and MD ATPase activities^24,37,38^. In solution, the stalk helices predominantly assume a low-affinity registration called the β registration^38,45^. However, after MT binding, the stalk helices slide into the high-affinity α registration^38,46^. In the presence of applied mechanical tension, dynein assumes the strong MT-binding α registry under backward load, while forward load induces the recently discovered γ registry with intermediate MT-binding strength^24^. The stalk assumes the β registry upon AAA1-ATP binding^24^, promoting dissociation of the MTBD from the MT.

Adding complexity to the interactions among AAA1, AAA3, dynein MTBDs, and external and intramolecular tension is evidence that AAA4 may have a regulatory role. When ATP hydrolysis by AAA4 is prevented, dynein processivity increases two-fold and MT-binding affinity increases five-fold^47^. However, blockage of AAA4-ATP hydrolysis reduces dynein velocity only slightly^47^, in contrast to prevention of AAA1-ATP or AAA3-ATP hydrolysis, which dramatically effect velocity (prevention of AAA1 hydrolysis makes the motor immobile)^47,48^. In addition, work on *Dictyostelium* dynein has shown that blockage of AAA4-ATP binding reduces MT-gliding velocity by ~60% while blockage of AAA3-ATP binding reduces velocity by ~95%^48^. It was therefore commonly assumed that AAA4 played a relatively minor role in regulating dynein’s mechanochemical stepping cycle, while AAA1 and AAA3 were the key regulators.

Here, we combine mutagenesis with single-molecule fluorescence and optical tweezers-based force measurements^24,49–52^ to demonstrate that AAA4 is a major regulator of dynein motility. We show that while blockage of AAA4-ATP hydrolysis reduces the speed of *S. cerevisiae* dynein motion only a minimal degree, preventing AAA4-ATP binding abolishes dynein motility completely. We demonstrate that this effect is due to AAA4 regulation of the linker, showing that nucleotide-binding to AAA4 is required for the linker to undock from the MD and perform its priming stroke. Through this mechanism, AAA4 also governs the buttress-mediated sliding of the stalk helices, enabling the stalk to slide from the γ to the β registration. We further elucidate the interplay between the three active AAA+ ATPase domains. We find that blocking AAA4-ATP binding induces the γ registry independent of the direction of applied tension if AAA1 is bound to ATP but irrespective of the nucleotide state of AAA3. However, when AAA4 is bound to ATP, the gating of AAA1 by AAA3 prevails, revealing that AAA4 gates the activities of AAA3 and explaining why the effect of preventing ATP hydrolysis by AAA4 on dynein velocity is so mild. Thus, AAA1, 3, and 4, the three active ATPases of the MD, work together to regulate dynein’s cyclic MT-interactions.

## Results

### AAA4-ATP binding is required for dynein motion and force generation

To determine the importance of AAA4 in regulating dynein motion, we used a single-molecule fluorescence motility assay^51–53^ to define the effects of ATP-binding mutations (K/A mutation in the Walker A motif^22,54^) and ATP-hydrolysis mutations (E/Q mutation in the Walker B motif^54,55^) in AAA3 and AAA4 on dynein motility. We introduced E/Q and K/A mutations in Dyn1_331kDa_, a minimal *S. cerevisiae* MD containing the linker, AAA+ ring, stalk, buttress and MTBD (this construct retains its motor activities^42,52,56^ and is equivalent to the *Dictyostelium discoideum* MD used in key biochemical studies^19,22,37,39^). We then dimerized the single-headed GFP-Dyn1_331kDa_ mutants using an antibody against the N-terminal GFP^24^ and studied the motors using total internal reflection fluorescence (TIRF) microscopy^51,52^.

**Fig. 2:**
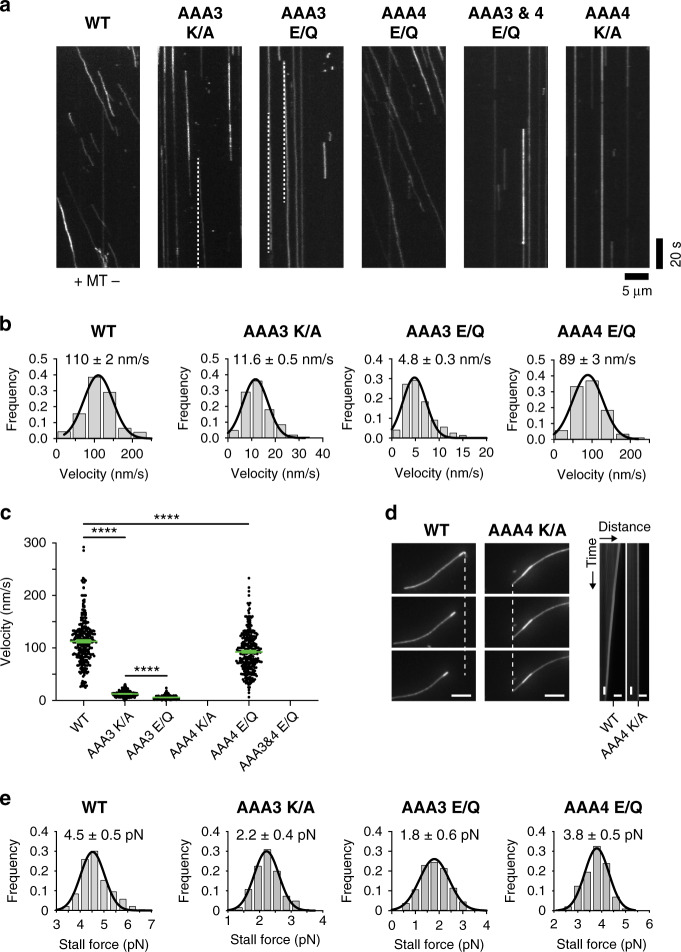
AAA4-ATP binding is essential for dynein motility. **a** Antibody-dimerized WT Dyn1_331kDa_ moves processively in the single-molecule TIRF assay. Diagonal lines in the kymograph represent dimerized molecules that are moving over time. Preventing ATP binding (K/A) or ATP hydrolysis (E/Q) by AAA3 dramatically slows down dynein motion (dashed white lines serve as visual guides to identify slow moving mutants). In contrast, preventing ATP hydrolysis by AAA4 only slightly reduces dynein speed. Strikingly, preventing ATP hydrolysis by both AAA3 and AAA4 or preventing AAA4-ATP binding only, completely abolishes dynein motion (experiments were repeated 3 and 5 times, respectively, with the same results). **b** Fitting the WT velocity histograms with a Gaussian fit (black line), returns a mean velocity of 110 ± 2 nm/s (±SEM; *N* = 284; obtained from *n* = 6 independent experiments). Analysis of the AAA3 mutants confirms the low velocities suggested by the kymographs: 11.6 ± 0.5 nm/s (±SEM; *N* = 243; *n* = 5) for the Dyn1_331kDa_ AAA3 K/A mutant and 4.8 ± 0.3 nm/s (*N* = 295; *n* = 6) for the Dyn1_331kDa_ AAA3 E/Q mutant. The Dyn1_331kDa_ AAA4 E/Q mutant moves with a mean velocity of 89 ± 3 nm/s (*N* = 307; *n* = 5). **c** Comparison of the WT GFP-Dyn1_331kDa_, AAA3 K/A GFP-Dyn1_331kDa_, AAA3 E/Q GFP-Dyn1_331kDa_, and AAA4 E/Q GFP-Dyn1_331kDa_ mean velocities (±SEM). Statistical significance was determined using an unpaired Welch’s *t* test (****p < 0.0001). **d** Kymograph analysis of TMR-labeled and polarity-marked MTs bound to a cover glass via surface-anchored WT Dyn1_331kDa_ and Dyn1_331kDa_ AAA4 K/A in the presence of 1 mM ATP. Left: image sequence showing the gliding of a MT with the bright minus-end lagging, revealing the minus-end-directed activity of WT Dyn1_331kDa_. Middle: image sequence of an immobile MT bound rigidly to the cover glass via the Dyn1_331kDa_ AAA4 K/A mutant, demonstrating that this mutant is able to bind MTs but incapable of gliding them. Right: kymographs of image sequences of 1,200 images each with representative images shown on the left. The depicted horizontal scale bars correspond to a distance of 5 μm and the vertical scale bars to a time period of 10 s. **e** Histogram of stall forces measured for homodimeric full-length WT GFP-Dyn1_471kDa_, AAA3 K/A GFP-Dyn1_471kDa_, AAA3 E/Q GFP-Dyn1_471kDa_, and AAA4 E/Q GFP-Dyn1_471kDa_ (mean ± SD). The number of events for each histogram: WT, 189; AAA3 K/A, 119; AAA3 E/Q, 130; and AAA4 E/Q, 189. Source data are provided as a Source Data file.

Using this approach, we first confirmed that blocking AAA3-ATP binding or hydrolysis significantly reduced velocities (11.6 ± 0.5 nm/s and 4.8 ± 0.3 nm/s) when compared to WT dynein (110 ± 2 nm/s) (Fig. 2a, b)^43,47,48,57^, while preventing ATP hydrolysis by AAA4 had only minor effects on dynein velocity^47^ (velocity reduced to 89 ± 3 nm/s), consistent with previous reports (Fig. 2a, b). In contrast, blocking ATP binding to AAA4 in the AAA4 K/A single mutant completely abolished dynein motion (as did blocking ATP hydrolysis by both AAA3 and AAA4 in the AAA3 E/Q + AAA4 E/Q double mutant) (Fig. 2a). Thus, ATP binding to AAA4 is required for dynein motility.

To further assess the effects of ATP-binding and/or ATP-hydrolysis mutations on dynein motion under load, we created homodimeric full-length dynein mutants carrying either the AAA3 E/Q, AAA3 K/A, or AAA4 E/Q mutations and used an optical tweezers setup^49,50,58^ to determine their force-generation capabilities (AAA4 K/A cannot generate motion and therefore cannot be tested in an optical trap). While WT yeast dynein generates 4.5 pN^59^ (Fig. 2e), the AAA3 E/Q and AAA3 K/A mutants exhibit ~60% and ~50% reduced stall forces (1.8 and 2.2 pN, respectively). Consistent with the minor effects on zero-load motility (Fig. 2a, b), the AAA4 E/Q mutant shows only a ~15% reduced stall force (Fig. 2e). The relative reductions in force generation of the AAA3 E/Q and AAA4 E/Q mutants (~60% and ~15%) compared to WT are comparable with the previous measurements on tail-truncated GST-dimerized mutant motors (~40% and ~20%, respectively)^47^ (note, however, that while the relative changes are comparable, the absolute forces reported for the GST-dimerized mutant motors are overestimated as a result of an unintended electronic low-pass filtering of the trapping data as we recently discussed^59^). These results confirm that preventing AAA3-ATP hydrolysis has a more dramatic effect on dynein’s ability to generate force than preventing ATP hydrolysis by AAA4. Thus, while our stall-force measurements on the AAA3-ATP and AAA4-ATP hydrolysis mutants alone would support a minor regulatory role for AAA4, our single-molecule TIRF studies on the AAA4-ATP binding mutant reveal that AAA4 has a previously unrecognized essential regulatory role.

### ATP binding to AAA4 is required for dynein’s priming stroke

The finding that blocking AAA4-ATP binding completely prevents dynein motion (Fig. 2a) surprised us, given the previous work on *Dictyostelium* dynein that showed that the single-headed AAA4 K/A mutant was able to power MT-gliding along coverslip surfaces at ~40% of the velocity of WT dynein^48^. In contrast, we did not observe MT-gliding by our single-headed *S. cerevisiae* AAA4 K/A mutant, while single-headed WT dynein moved MTs smoothly (Fig. 2d). We next sought to understand how the AAA4 mutation prevents dynein motion. For dynein to take a forward step, ATP must bind to AAA1, which causes dissociation of the linker from the rear head AAA5 domain^17,60^ (Fig. 1b, c), transition of the stalk helices from the γ registry into the weak MT-binding β registry^24^, followed by MT release. Linker undocking from AAA5 and subsequent rear head dissociation from the MT are required for the linker to complete its priming stroke^24^. We hypothesized that AAA4 might regulate linker undocking from AAA5, either directly, due to its proximity to the linker docking site, or indirectly, through allosterically regulating AAA1 and/or AAA3 activity.

**Fig. 3:**
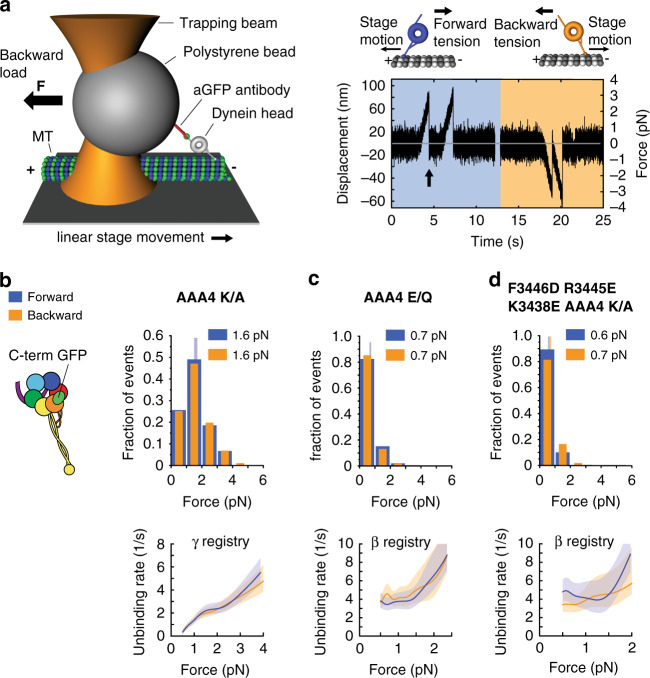
AAA4-ATP binding controls the linker undocking and the transition from the γ registry to the β registry. **a** (Left) A polystyrene bead bearing a dynein motor is held in an optical trap as the microscope stage sweeps back and forth parallel to a MT (not to scale). (Right) Position (force) vs. time for the AAA4 K/A Dyn1_331 kDa_-GFP mutant in the presence of 1 mM ATP. Orange and blue shaded areas show periods of applied backward and forward tension, respectively (loading rate: 5.6 pN/s; *k* = 0.036 pN/nm, *v*_stage_ = 156 nm/s). After the motor binds the MT, it pulls the bead out of the trap. Force on the motor increases until the dynein-MT bond ruptures at the “unbinding force” (arrow), here ∼3 pN. **b** (Left) Schematic of dynein with GFP fused to the C-terminus. (Right) Normalized histograms of primary forward and backward unbinding forces of the AAA4 K/A Dyn1_331 kDa_-GFP mutant in the presence of 1 mM ATP. The mean values are noted. Tall vertical bands represent 95% CIs of the means (forward: [1.5, 1.7] pN, backward: [1.5, 1.7] pN), which were estimated by bootstrapping 4000 samples. (Bottom) Unbinding rate vs. force derived from the data above. The shaded areas are 95% CIs for the mean rates, which were also estimated by bootstrapping. **c** Same as **b**, but for the AAA4 E/Q mutant (95% CIs [0.64, 0.70] and [0.64, 0.71] pN). **d** Same as in **b**, but for the F3446D R3445E K3438E AAA4 K/A-Dyn1_331kDa_-GFP mutant (95% CIs [0.63, 0.7] and [0.65, 0.72] pN). The number of events in the forward and backward directions: (**b**) (269, 278), (**c**) (409, 429), and (**d**) (236, 151). Source data are provided as a Source Data file.

Our previous work demonstrated that linker undocking from AAA5 is required for the transition from the γ registry into the β registry^24^ (Fig. 1c). We therefore tested how the AAA4 K/A and AAA4 E/Q mutations affected stalk registry under load by determining dynein-MT binding strengths as a function of the direction of applied tension using optical tweezers, as we have done before^24,42^. In this assay, a polystyrene bead bearing a dynein MD is held in an optical trap as the microscope stage sweeps back and forth parallel to a MT (Fig. 3a, left). The motor binds the MT, pulling the bead out of the trap center. The force on the motor increases until the dynein–MT bond ruptures at the “unbinding force” (Fig. 3a, right). Converting the measured unbinding forces into force-dependent unbinding rates (using a theoretical framework introduced by Olga Dudko and colleagues^61^ and further adapted by us to take into account the compliance of the motor^24^) then allows the assignment of stalk registrations. For example, in the absence of nucleotides (apo state), a WT motor domain assumes the α registration (strong MT binding state) under backward load, indicated by an average unbinding force of ~3 pN, and assumes the γ registration under forward load with an average unbinding force of ~1.6 pN^24,42^. In contrast, if the motor is locked in the AAA1 ATP-bound state (AAA1 E/Q mutation), the motor assumes the β registration in both directions, exhibiting an average unbinding force of ~0.7 pN in both directions^24,42^ (as previously discussed^24^, the measured forces dependent on the chosen experimental conditions including the loading rate that the dynein-MT bound experiences). We therefore performed unbinding force measurements in the presence of ATP using dynein constructs bearing the AAA4-ATP binding or hydrolysis mutation, and hypothesized that if ATP binding to AAA4 is required for linker undocking from AAA5, the stalk would also be unable to transition from the γ to the β registration.

We used Dyn1_331kDa_ constructs bearing a C-terminal GFP (Fig. 1a) for the attachment to anti-GFP antibody coated optical trapping beads (Fig. 3a). Dyn1_331kDa_-GFP contains the linker, AAA+ ring, stalk, buttress and MTBD (Fig. 1a), and the linker of this construct can freely assume the pre-powerstroke and post-powerstroke states in the absence of applied tension (Fig. 1b, c). Under C-terminus-applied tension, ATP binding to AAA1 of a WT construct causes dissociation of the linker and the expected transition of the stalk helices from the γ to the β registry^24^ (Table 1).

**Table 1:** Relation of nucleotide condition, pulling position, AAA+ ATPase mutations and stalk-helix registrations.

Nucleotide condition*	Dyn1_331kDa_ construct	Stalk registry backward load/forward load	Summary
	**Pulling position: C-terminus**		
Apo	WT^42^	α/γ	ATP binding to WT dynein is sufficient to induce the β registration under C-terminal tension.
ATP	WT^42^	β/β	
ATP	AAA4 E/Q	β/β	Gating of AAA1 by AAA3 prevails if AAA4 is bound to ATP.
ATP	AAA3 E/Q^42^	α/γ	
ATP	AAA3 E/Q + AAA4 E/Q	α/γ	
ATP	AAA4 K/A	γ/γ	The effects of blocking AAA4-ATP binding on stalk registry require functional interactions between the linker and AAA5
ATP	F3446D R3445E K3438E AAA4 K/A	β/β	
	**Pulling position: N-terminus**		
Apo	WT^24,42^	α/γ	In the presence of ATP and N-terminal tension, dynein samples through strong, intermediate and weak MT-binding states as a result of tension-induced effects on the AAA1 nucleotide-binding site^42^.
ATP	WT^42^	β, α /β, γ	
ATP	AAA1 E/Q^42^	β/β	The β registration can only be induced when AAA4 is bound to nucleotide.
ATP	AAA1 E/Q + AAA3 E/Q^42^	β/β	
ATP	AAA1 E/Q + AAA4 E/Q	β/β	
ATP	AAA1 E/Q + AAA3 E/Q + AAA4 E/Q	β/β	
ATP	AAA1 E/Q + AAA3 E/Q + AAA4 K/A	γ/γ	
ATP	AAA1 E/Q + AAA3 K/A^42^	α/γ	Even when the AAA3-based gate on AAA1 is closed, the α registration can only be induced if AAA4 is bound to nucleotide.
ATP	AAA1 E/Q + AAA3 K/A + AAA4 E/Q	α/γ	
ATP	AAA1 E/Q + AAA3 K/A + AAA4 K/A	γ/γ	
ATP	AAA1 E/Q + AAA4 K/A	γ/γ	
ATP	AAA1 K/A^42^	α/γ	Blocking AAA1-ATP binding has a dominant effect on stalk registries and induces the α registry under backward load and the γ registry under forward load irrespective of the nucleotide state of AAA4.
ATP	AAA1 K/A + AAA4 E/Q	α/γ	
ATP	AAA1 K/A + AAA4 K/A	α/γ	

*The concentration of ATP is 1 mM.

We determined the MT-binding strengths of both AAA4 mutants under C-terminus applied tension and compared their behavior with that of WT Dyn1_331kDa_ with the stalk helices cross-linked (CL) into different registries (cross-linked constructs were generated previously by introducing cysteines in the outgoing (CC1) and return (CC2) α-helices of the dynein stalk for reversible disulfide cross-linking, in order to lock Dyn1_331kDa_ into fixed stalk registries^24^). In the presence of 1 mM ATP, the AAA4 K/A mutant with the C-terminal GFP exhibited indistinguishable behavior from the Dyn1_331kDa_ mutant with the cross-linked γ registry, Dyn1_331kDa_-γ CL (Fig. 3b and Tables 1, 2). In contrast, the AAA4 E/Q mutant in the presence of 1 mM ATP behaved similarly to Dyn1_331kDa_-β CL, the Dyn1_331kDa_ mutant with the cross-linked β registry (Fig. 3c and Tables 1, 2). These results demonstrate that ATP binding to AAA4 is required for transition of the stalk helices into the β-registry, supporting the hypothesis that ATP binding to AAA4 is required for linker dissociation from AAA5 and subsequent transition of the stalk helices into the β-registry.

**Table 2:** Results of statistical comparisons for various measured unbinding force histograms.

Experiment 1 (pN), mean [CI]	Experiment 2 (pN), mean [CI]	*p*_m_
AAA4 K/A Dyn1_331kDa_-GFP 1 mM ATP forward 1.6 [1.5, 1.7]	Dyn1_331kDa_-γ CL apo forward^24^ 1.6 [1.5, 1.7]	<0.75
AAA4 K/A Dyn1_331kDa_-GFP 1 mM ATP backward 1.6 [1.5, 1.7]	Dyn1_331kDa_-γ CL apo backward^24^ 1.6 [1.6, 1.7]	<0.65
AAA4 E/Q Dyn1_331kDa_-GFP 1 mM ATP forward 0.7 [0.6, 0.7]	Dyn1_331kDa_-β CL apo forward^24^ 0.7 [0.6, 0.7]	<0.2
AAA4 E/Q Dyn1_331kDa_-GFP 1 mM ATP backward 0.7 [0.6, 0.7]	Dyn1_331kDa_-β CL apo backward^24^ 0.7 [0.7, 0.8]	<0.4
F3446D R3445E K3438E AAA4 K/A-Dyn1_331kDa_-GFP 1 mM ATP forward 0.6 [0.6, 0.7]	Dyn1_331kDa_-β CL apo forward^24^ 0.7 [0.6, 0.7]	<0.22
F3446D R3445E K3438E AAA4 K/A-Dyn1_331kDa_-GFP 1 mM ATP backward 0.7 [0.7, 0.7]	Dyn1_331kDa_-β CL apo backward^24^ 0.7 [0.7, 0.8]	<0.45
AAA1 E/Q + AAA4 E/Q-GFP-Dyn1_331kDa_ 1 mM ATP forward 0.7 [0.6, 0.7]	Dyn1_331kDa_-β CL apo forward^24^ 0.7 [0.6, 0.7]	<0.83
AAA1 E/Q + AAA4 E/Q-GFP-Dyn1_331kDa_ 1 mM ATP backward 0.7 [0.6, 0.7]	Dyn1_331kDa_-β CL apo backward^24^ 0.7 [0.7, 0.8]	<0.38
AAA1 E/Q + AAA3 E/Q + AAA4 E/Q-GFP-Dyn1_331kDa_ 1 mM ATP forward 0.7 [0.6, 0.7]	Dyn1_331kDa_-β CL apo forward^24^ 0.7 [0.6, 0.7]	<0.83
AAA1 E/Q + AAA3 E/Q + AAA4 E/Q-GFP-Dyn1_331kDa_ 1 mM ATP backward 0.7 [0.6, 0.7]	Dyn1_331kDa_-β CL apo backward^24^ 0.7 [0.7, 0.8]	<0.3
AAA1 E/Q + AAA4 K/A-GFP-Dyn1_331kDa_ 1 mM ATP forward 1.6 [1.4, 1.7]	Dyn1_331kDa_-γ CL apo forward^24^ 1.6 [1.5, 1.7]	<0.96
AAA1 E/Q + AAA4 K/A-GFP-Dyn1_331kDa_ 1 mM ATP backward 1.7 [1.5, 1.9]	Dyn1_331kDa_-γ CL apo backward^24^ 1.6 [1.6, 1.7]	<0.66
AAA1 E/Q + AAA3 E/Q + AAA4 K/A-GFP-Dyn1_331kDa_ 1 mM ATP forward 1.5 [1.4, 1.7]	Dyn1_331kDa_-γ CL apo forward^24^ 1.6 [1.5, 1.7]	<0.62
AAA1 E/Q + AAA3 E/Q + AAA4 K/A-GFP-Dyn1_331kDa_ 1 mM ATP backward 1.6 [1.5, 1.7]	Dyn1_331kDa_-γ CL apo backward^24^ 1.6 [1.6, 1.7]	<0.33
AAA1 E/Q + AAA3 K/A + AAA4 K/A-GFP-Dyn1_331kDa_ 1 mM ATP forward 1.5 [1.4, 1.6]	Dyn1_331kDa_-γ CL apo forward^24^ 1.6 [1.5, 1.7]	<0.32
AAA1 E/Q + AAA3 K/A + AAA4 K/A-GFP-Dyn1_331kDa_ 1 mM ATP backward 1.5 [1.5, 1.7]	Dyn1_331kDa_-γ CL apo backward^24^ 1.6 [1.6, 1.7]	<0.53
AAA1 K/A + AAA4 K/A-Dyn1_331kDa_-GFP 1 mM ATP forward 1.6 [1.5, 1.7]	Dyn1_331kDa_-γ CL apo forward^24^ 1.6 [1.5, 1.7]	<0.63
AAA1 K/A + AAA4 K/A-Dyn1_331kDa_-GFP 1 mM ATP backward 2.7 [2.4, 3]	Dyn1_331kDa_-α CL apo backward^24^ 2.7 [2.5, 3]	<0.8
AAA1 K/A + AAA4 E/Q-GFP-Dyn1_331kDa_ 1 mM ATP forward 1.6 [1.5, 1.7]	Dyn1_331kDa_-γ CL apo forward^24^ 1.6 [1.5, 1.7]	<0.63
AAA1 K/A + AAA4 K/A-GFP-Dyn1_331kDa_ 1 mM ATP backward 2.7 [2.4, 3]	Dyn1_331kDa_-α CL apo backward^24^ 2.7 [2.5, 3]	<0.79
AAA1 E/Q + AAA3 K/A + AAA4 E/Q-GFP-Dyn1_331kDa_ 1 mM ATP forward 1.6 [1.5, 1.6]	Dyn1_331kDa_-γ CL apo forward^24^ 1.6 [1.5, 1.7]	<0.84
AAA1 E/Q + AAA3 K/A + AAA4 E/Q-GFP-Dyn1_331kDa_ 1 mM ATP backward 2.6 [2.4, 2.8]	Dyn1_331kDa_-α CL apo backward^24^ 2.7 [2.5, 3]	<0.53
AAA3 E/Q + AAA4 E/Q-Dyn1_331kDa_-GFP 1 mM ATP forward 1.6 [1.5, 1.7]	Dyn1_331kDa_-γ CL apo forward^24^ 1.6 [1.5, 1.7]	<0.8
AAA3 E/Q + AAA4 E/Q-Dyn1_331kDa_-GFP 1 mM ATP backward 2.6 [2.4, 2.9]	Dyn1_331kDa_-α CL apo backward^24^ 2.7 [2.5, 3]	<0.7

To directly test whether the inability of the AAA4 K/A mutant stalk helices to slide was indeed due to prevention of linker undocking from AAA5, we mutated three conserved residues in the AAA5 linker-docking site that have been previously shown to be critical for the docking of the linker to AAA5^62^ (F3446D, R3445E, and K3438E) (Fig. 1c). We anticipated that this mutant would assume the β registry despite having AAA4-ATP binding blocked. Indeed, the F3446D R3445E K3438E AAA4 K/A Dyn1_331kDa_ quadruple mutant exhibited statistically indistinguishable behavior from the Dyn1_331kDa_-β CL mutant (Fig. 3d and Tables 1, 2), confirming our hypothesis that blocking AAA4-ATP binding prevents transition into the β-registry by preventing linker undocking.

### AAA4-based block on linker undocking is independent of AAA3

Previous work demonstrates that ATP binding to AAA1 is required for linker undocking^23^ and that AAA3 gates AAA1 activity, so that AAA1-ATP binding induces undocking of the linker and the weak MT-binding β registration only when AAA3 is in the post-hydrolysis state^42,43^. However, when tension is applied to the linker, ATP binding to AAA3 is sufficient to “open” the gate^42^. The conclusion that AAA3 affects the linker indirectly via AAA1 is supported by the MT-binding behavior of the AAA3 E/Q mutant: blocking AAA3-ATP hydrolysis yields the γ registry under forward load and the α registry under backward load^24,42^, a behavior identical to that of the AAA1-ATP binding (AAA1 K/A) mutant and the WT motor in the apo state^24,42^ (Table 1). In contrast, blocking AAA4-ATP binding yields the γ registry in both directions (Fig. 3a, b). This indicated that unlike AAA3, which exerts regulatory control of linker undocking indirectly through AAA1, ATP binding to AAA4 regulates linker undocking and stalk registry changes through a different pathway. We hypothesized, given AAA4’s proximity to the linker docking site at AAA5, that AAA4 may regulate linker undocking and stalk registry changes directly.

To provide further evidence that the block imposed on linker undocking by AAA4 is direct and to elucidate the hierarchy between the AAA4 K/A-induced block on the linker transition from a docked to an undocked state and AAA1 and AAA3 function, we employed novel double and triple AAA + ATPase mutants.

**Fig. 4:**
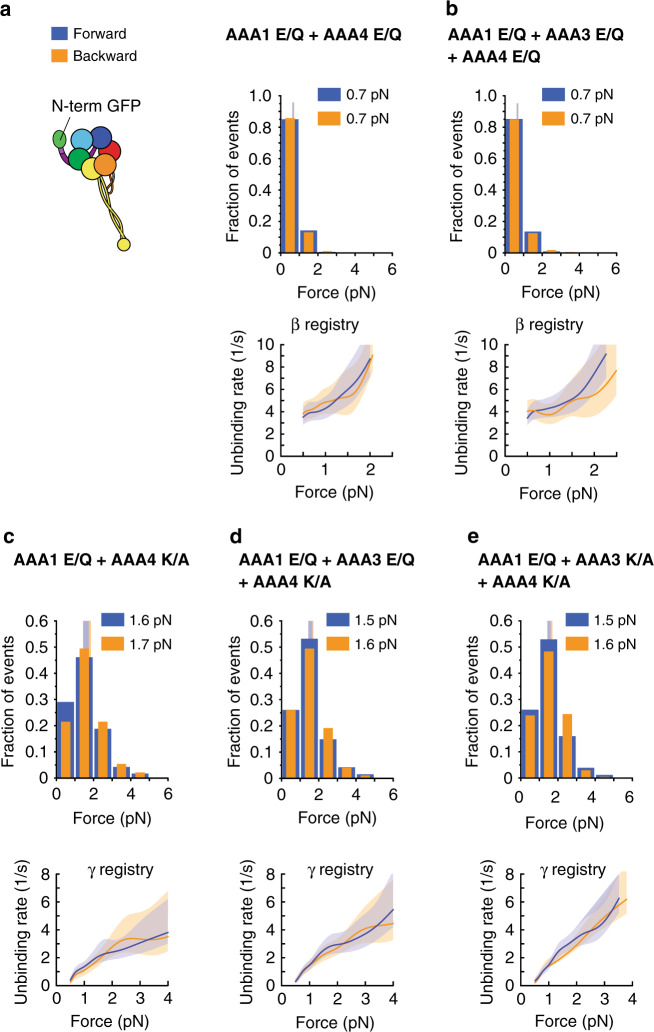
Blocking AAA4-ATP binding induces the γ registry irrespective of the AAA3 nucleotide state. **a** (Left) Schematic of dynein with GFP fused to the N-terminus. (Right) Histogram of forward (blue) and backward (orange) unbinding forces for the AAA1 E/Q + AAA4 E/Q-GFP-Dyn1_331kDa_ double mutant measured in the presence of 1 mM ATP. The respective mean values are noted. The vertical bands represent 95% CIs for the means (forward: [0.6, 0.7] pN, backward [0.6, 0.7] pN). (Bottom) Unbinding rate vs. force derived from the data above. The shaded areas are the 95% CIs for the mean rates, which were estimated by bootstrapping. **b** Same as in **a**, but for the AAA1 E/Q + AAA3 E/Q + AAA4 E/Q-GFP-Dyn1_331kDa_ triple mutant (95% CIs [0.6, 0.7] and [0.6, 0.7] pN). **c** Same as in **a**, but for the AAA1 E/Q + AAA4 K/A-GFP-Dyn1_331kDa_ double mutant (95% CIs [1.4, 1.7] and [1.5, 1.9] pN). **d** Same as in **a**, but for the AAA1 E/Q + AAA3 E/Q + AAA4 K/A-GFP-Dyn1_331kDa_ triple mutant (95% CIs [1.4, 1.7] and [1.5, 1.7] pN). **e** Same as in **a**, but for the AAA1 E/Q + AAA3 K/A + AAA4 K/A-GFP-Dyn1_331kDa_ triple mutant (95% CIs [1.4, 1.6] and [1.5, 1.7] pN). The number of events in the forward and backward directions: (**a**) (321,358), (**b**) (419, 382), (**c**) (117, 93), (**d**) (188,172), and (**e**) (257, 205). Source data are provided as a Source Data file.

We first confirmed that the AAA4 K/A-induced block on linker undocking was independent of AAA3. As mentioned, under linker-applied tension, the binding of ATP to AAA3 opens the gate and AAA1-ATP binding induces the β registry^24,42^. Thus, in the presence of 1 mM ATP, the AAA1 E/Q single mutant and the AAA1 E/Q + AAA3 E/Q double mutant assume the β registry in both directions, while the AAA1 K/A single mutant and the AAA1 E/Q + AAA3 K/A double mutant assume the γ registry under forward load and the α registry under backward load^24,42^ (Table 1). As ATP binding to AAA4 is required for the transition into the β registry (Fig. 3b, c), we anticipated that the AAA1 E/Q + AAA4 E/Q double mutant and the AAA1 E/Q + AAA3 E/Q + AAA4 E/Q triple mutant would assume the β registry in the presence of 1 mM ATP, while blocking AAA4-ATP binding in the double mutant (AAA1 E/Q + AAA4 K/A) and the triple mutant (AAA1 E/Q + AAA3 E/Q + AAA4 K/A) would prevent reduced MT binding and induce the γ registry in both directions, in spite of the AAA3 gate being open and AAA1 nucleotide bound. As expected, the AAA1 E/Q + AAA4 E/Q double mutant and the AAA1 E/Q + AAA3 E/Q + AAA4 E/Q triple mutant assumed the β registry in the presence of 1 mM ATP (Fig. 4a, b and Tables 1, 2), while the AAA1 E/Q + AAA4 K/A double mutant and the AAA1 E/Q + AAA3 E/Q + AAA4 K/A triple mutant assumed the γ registry in both directions (Fig. 4c, d and Tables 1, 2).

To test whether blocking ATP binding to AAA4 was also able to induce the γ registry when AAA3 was nucleotide-free (when the gate on AAA1 is closed), we explored the MT-binding behavior of the AAA1 E/Q + AAA3 K/A + AAA4 K/A triple mutant. As expected, this triple mutant also assumed the γ registry in the presence of 1 mM ATP in both directions (Fig. 4e and Tables 1, 2). Thus, the ability of AAA4’s nucleotide free state to block changes in MT-binding strength is independent of whether the AAA3 gate is closed or open. In addition, the fact that the AAA1 E/Q + AAA4 K/A double mutant assumed the γ registry irrespective of the direction of applied tension (Fig. 4d) demonstrates that the AAA4 K/A mutation also blocked linker undocking even when AAA3 assumed a post-ATP-hydrolysis state (AAA3 of this mutant can bind and hydrolyze ATP and reach the ADP⋅Pi transition state for some fraction of the dynein cycle^43^, which is sufficient to open the gate on AAA1 so that the AAA1 E/Q single mutant assumes the β registry at 1 mM ATP^24,42^). Therefore, AAA4’s block on linker undocking is independent of the nucleotide state of AAA3.

### AAA4-based regulation of MT-binding strength is dependent on the nucleotide state of AAA1

As discussed, blocking AAA4-ATP binding induces the γ registry in both MT directions (Fig. 3a, b). In contrast, WT dynein in the absence of nucleotides (apo) and the AAA1 K/A single mutant in the presence of ATP both assume the α registry under backward load^24,42^ (Table 1). Therefore, blocking ATP binding to AAA4 alone has a distinct effect on stalk registry from blocking nucleotide binding to both AAA1 and AAA4, or blocking ATP binding to AAA1 alone. This suggests that while AAA4 regulation of stalk registry is independent of the nucleotide-binding state of AAA3, it is dependent upon the nucleotide-binding state of AAA1.

**Fig. 5:**
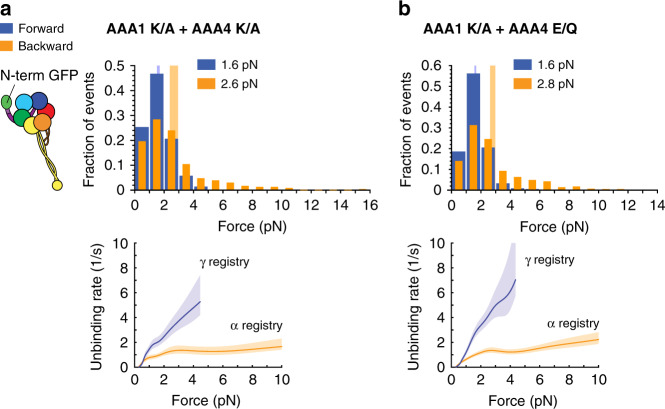
The effects of AAA4 on dynein-MT binding require AAA1-ATP binding. **a** (Left) Schematic of dynein with GFP fused to the N-terminus. (Right) Histogram of forward (blue) and backward (orange) unbinding forces for the AAA1 K/A + AAA4 K/A-GFP-Dyn1_331kDa_ double mutant measured in the presence of 1 mM ATP. The respective mean values are noted. The vertical bands represent 95% CIs for the means (forward: [1.5, 1.7] pN, backward [2.4, 3] pN). (Bottom) Unbinding rate vs. force derived from the data above. The shaded areas are the 95% CIs for the mean rates, which were estimated by bootstrapping. **b** Same as in *a*, but for the AAA1 K/A + AAA4 E/Q-GFP-Dyn1_331kDa_ double mutant (95% CIs [1.6, 1.7] and [2.6, 3] pN). The number of events in the forward and backward directions: (**a**) (276,229) and (**b**) (575, 569). Source data are provided as a Source Data file.

To confirm this, we generated the AAA1 K/A + AAA4 E/Q and the AAA1 K/A + AAA4 K/A double mutants and performed unbinding-force measurements. As expected, both double mutants assume the α registry under backward load and the γ registry under forward load in the presence of ATP (Fig. 5a, b and Tables 1, 2). This implies that the effect of ATP binding to AAA4 on linker undocking is secondary to the regulation of linker undocking and stalk sliding by AAA1, since blockade of ATP-binding to AAA4 only impacts stalk registry when AAA1 is nucleotide bound.

### AAA3 controls ATP-induced, AAA1-mediated MT release when AAA4 is bound to ATP

Our work demonstrates that AAA4 regulation of linker undocking is independent of AAA3’s nucleotide state, but dependent on the nucleotide state of AAA1 (whether the AAA3 gate was opened or closed, the stalk helices assumed the γ registry if AAA4 ATP-binding was blocked, but only if AAA1 is ATP bound). The observation that blocking AAA4-ATP hydrolysis has only a minor effect on dynein function (Fig. 2) suggests that AAA4 no longer exerts a dominant role once bound to ATP. Therefore we hypothesized that if AAA4 is nucleotide bound, AAA3 assumes its role in controlling AAA1 function^42,43^.

**Fig. 6:**
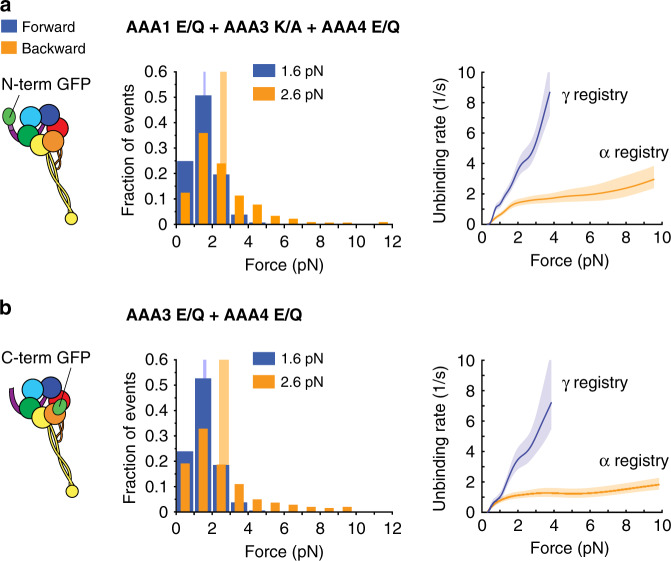
ATP binding to AAA4 is not sufficient to overcome the AAA3-induced block on AAA1. **a** Schematic of dynein with GFP fused to the N-terminus (left). Histogram of forward (blue) and backward (orange) unbinding forces for the AAA1 E/Q + AAA3 K/A + AAA4 E/Q-GFP-Dyn1_331kDa_ triple mutant measured under N-terminal tension in the presence of 1 mM ATP (middle). The respective mean values are noted. The vertical bands represent 95% CIs for the means (forward: [1.5, 1.6] pN, backward [2.4, 2.8] pN). (Right) Unbinding rate vs. force derived from the data on the left. The shaded areas are 95% CIs for the mean rates, which were estimated by bootstrapping. **b** Same as in *a*, but for the AAA3 E/Q + AAA4 E/Q-Dyn1_331kDa_-GFP double mutant measured under C-terminal tension (95% CIs [1.5, 1.7] and [2.4, 3] pN). The number of events in the forward and backward directions: (**a**) (546,451) and (**b**) (279, 234). Source data are provided as a Source Data file.

To test this hypothesis, we determined the unbinding behavior of the AAA1 E/Q + AAA3 K/A + AAA4 E/Q triple mutant with the N-terminal GFP. Under N-terminus-applied tension, blocking AAA3-ATP binding closes the gate on AAA1 (AAA1-ATP binding cannot induce MT release) and, as previously stated, results in the γ registry under forward load and the α registry under backward load^24,42^ (Table 1). As expected, we found that the AAA1 E/Q + AAA3 K/A + AAA4 E/Q triple mutant in the presence of 1 mM ATP assumed the γ registry under forward load and the α registry under backward load (Fig. 6a and Tables 1, 2). In addition, the AAA3 E/Q + AAA4 E/Q double mutant with the C-terminal GFP (under C-terminus-applied tension, blocking AAA3-ATP hydrolysis closes the gate on AAA1^42^; Table 1) also assumed the γ registry under forward load and the α registry under backward load in the presence of 1 mM ATP (Fig. 6b and Tables 1, 2). Thus, when AAA4 was ATP bound, AAA3 gates the activity of AAA1. Therefore, both AAA4 and AAA3 regulate dynein-MT binding strength, and both their regulatory mechanisms are dependent upon the nucleotide state of AAA1. This dual dominance underscores the intricate complexity and convoluted interactions among dynein’s primary and regulatory AAA + domains.

## Discussion

Previous studies suggested that AAA4 has only a minor role in dynein function. Here, we identify AAA4 as a key regulator of dynein motion, demonstrating that ATP binding to AAA4 is necessary for linker undocking and subsequent stalk-helices sliding and MT release. We further elucidate the hierarchy of regulation of MT-binding strength by the active AAA+ ATPase domains, demonstrating that nucleotide binding to AAA4 gates the regulatory function of AAA3, which in turn gates AAA1. While AAA3 exerts influence on MT-binding strength through an entirely allosteric mechanism via AAA1, our results suggest that AAA4 regulation of linker undocking and stalk sliding is more complex. The unique stalk-registry profile assumed by the AAA4 K/A mutant (the γ-registry under forward and backward load) suggests that nucleotide binding to AAA4 may directly influence linker conformation. At the same time, regulation of dynein-MT binding strength by AAA4 is dependent on the nucleotide state of AAA1: ATP binding to AAA4 alone has no effect on stalk registry unless AAA1 is also bound to nucleotide. These findings underscore the intricate complexity and convoluted interactions among dynein’s primary regulatory AAA+ domains in dynein’s mechanochemical cycle.

We recently reported that the linker controls the buttress-mediated sliding of the stalk helices to alter the MT-binding strength of the two MTBDs within dynein^24^. These past results combined with the data presented here suggest that AAA4 has an essential function in controlling dynein’s cyclic MT interactions through regulation of linker undocking and subsequent stalk-helices sliding. This conclusion appears to contradict a previous study on *Dictyostelium* dynein where it was reported that the AAA4 K/A mutation reduced MT-gliding activity by only 60%^48^. While it is possible that *Dictyostelium* and *S. cerevisiae* dynein behave differently in the mutant background, future studies will be required to resolve this discrepancy and to show whether AAA4 of mammalian dynein has the same function as AAA4 of *S. cerevisiae* dynein, but mutations in AAA4 that cause malformation of cortical development reinforce the importance of AAA4 in dynein mechanochemistry^9^.

Our single-molecule TIRF studies and the work of others^47^ have shown that the homodimeric AAA4 E/Q mutant moves processively at ~80% of the velocity of WT dynein (Fig. 2a, b). As the AAA4 E/Q mutant with the C-terminal GFP assumes the β registration in the presence of ATP (Fig. 3c), this result appears to be in conflict with our previous work that demonstrated that the dimerized Dyn1_331kDa_-β CL construct with the cross-linked β registry cannot move^24^. However, while Dyn1_331kDa_-β CL construct and the AAA4 E/Q mutant with the C-terminal GFP only assume the β registry, the AAA4 E/Q mutant with the N-terminal GFP can switch between different registrations depending on the nucleotide state of AAA1 and AAA3 (i.e., when tension is applied via the N-terminal linker) (Figs. 4 and 5). Together with our previous work that demonstrated that WT dynein with a C-terminal GFP assumes the β registry in the presence of ATP while WT dynein with an N-terminal GFP shows an unbinding behavior that reflects transitions through different stalk registrations^42^ (Table 1), this suggests that tension applied via the linker facilitates the motor’s progression through its mechanochemical cycle, and explains why the homodimeric AAA4 E/Q mutant would be able to move processively. This conclusion is supported by the work by Clearly and co-workers^56^ and the work of our lab^42^, which has shown that tension applied to the linker gates the ATP-dependent release of dynein from MTs. As the AAA4 E/Q mutant motor domains are linked together via the N-terminal GFPs (using an antibody against GFP, Fig. 2a, b) and as the mutant motor moves processively, this indeed suggests that the AAA4 E/Q mutant can assume different registrations (such as the α registration under backward load when AAA1 assumes the post-hydrolysis state, Fig. 5) if sufficient tension develops during the two-head bound state, while the Dyn1_331kDa_-β CL construct cannot.

Our single-molecule TIRF studies further revealed that the AAA3 E/Q + AAA4 E/Q double mutant does not move (Fig. 2), which can be explained by the MT-binding behavior of the mutant. Our unbinding-force studies showed that under C-terminus applied tension, AAA3 E/Q + AAA4 E/Q-Dyn1_331kDa_-GFP assumes the γ registry under forward load and the α registry under backward load in the presence of 1 mM ATP (Fig. 6b). Because both registrations can only be assumed when the linker is docked, the data suggest that the linker is always docked and that the priming stroke never occurs, and thus the motor cannot move. The Dyn1_331kDa_-α CL mutant still moves slowly^24^, which excludes the possibility that the ATP-insensitive MT-binding strength of the AAA3 E/Q + AAA4 E/Q double mutant accounts for its immobility. Intriguingly, the linker of the AAA3 E/Q mutant has also been shown to be locked in the post-powerstroke state in the absence of a load^63^, but the dimerized AAA3 E/Q mutant moves slowly (Fig. 2a, b). This implies that the linker of the AAA3 E/Q mutant can detach under tension and undergo a priming stroke—albeit less efficiently—whereas the AAA3 E/Q + AAA4 E/Q double mutant cannot. Therefore, the interaction strength between the linker and AAA5 could be stronger when ATP hydrolysis is prevented in both regulatory ATPase domains. The observation that the dimerized AAA4 K/A mutant is immotile further supports the premise that blocking AAA4-ATP binding could result in a stabilized, docked linker conformation.

The finding that blocked AAA4-ATP binding induced the γ registry independent of both the direction of applied tension and the nucleotide state of AAA3 is intriguing because it suggests that AAA4 can prevent tension-induced and nucleotide-induced changes in the stalk helices. Our data with the F3446D R3445E K3438E AAA4 K/A quadruple mutant showed that the effects of AAA4 on the stalk registry required functional interactions between the linker and AAA5 (Fig. 3d). However, our previous work has also demonstrated that the α registry requires the linker to dock to AAA5 in the apo state and that despite the docked state, directional tension can shift the registries interchangeably between α and γ^24,42^. Blocking AAA4-ATP binding locks the γ registry in both directions and prevents a change to the α registry under backward load, implying that the linker docks to AAA5 in a way that differs from the docked state required for the α registry. Indeed, the crystal structures in the absence of a nucleotide^62^ (Fig. 1b) and in the presence of ADP^21^ reveal two distinct post-powerstroke positions. In the apo state, the linker is positioned above the large domain of AAA5 (AAA5L)^62^. In the ADP state, the linker is positioned between AAA5L and the large domain of AAA4 (AAA4L)^21^. Direct linker interaction with AAA4, especially when AAA1 is bound to ATP and AAA4 is nucleotide-free, (Fig. 4d), remains to be shown. Solving the crystal structure of the AAA1 E/Q + AAA4 K/A double mutant (Fig. 4d) or the AAA1 E/Q + AAA3 E/Q + AAA4 K/A triple mutant (Fig. 4b) in the presence of ATP would help to evaluate this possibility.

Our results in Figs. 5 and 6 exemplified again the unique bond that dynein forms with a MT under backward load when the stalk helices assume the α registry. In agreement with our previous work^24,42^, the α registry caused dynein to exhibit “slip-ideal” bonding with the MTs for forces up to ~5–6 pN. Faster unbinding was observed for backward forces up to ∼2 pN and a constant, force-independent unbinding rate occurred for greater backward forces (Fig. 5). Dynein exhibited slip-bonding up to 4 pN when it assumed the γ registry^24^ (Fig. 3, 4). Noting that the movement of full-length *S. cerevisiae* dynein (Dyn1_471kDa_) ceases at 4.5 pN^59^ (“stall force”), these bond behaviors capture the most relevant force range of dynein when the motor is actively moving forward or stalling (when dynein is forced to move backward, larger forces can occur^64^).

While a recent study modeled the apo-state bond behavior of a single *S. cerevisiae* dynein head as an asymmetric slip bond for forces up to 14 pN^65^, a closer look at the measured data shows an excellent agreement with our results for forces up to ~5 pN, including the slip bonding up to ~2 pN and an ideal bonding up to ~5 pN under backward load. Our data also suggest an increasing unbinding rate above ~5 pN so that the bond appears best described by an overall slip-ideal-slip behavior for the 0–10 pN force range (Figs. 5 and 6), but the rates above ~5 pN that Ezber et al.^65^ reported are somewhat larger than ours. While the differences at larger forces could result from the different unbinding-force assays used^24^, future studies are necessary to accurately determine how the dynein-MT bond behaves under super-stall forces.

**Fig. 7:**
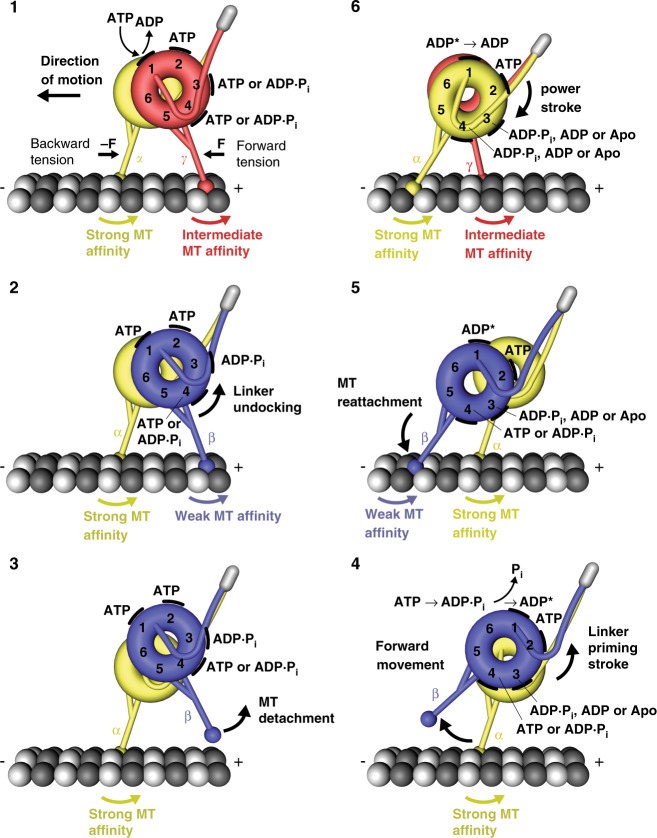
Model for the mechanochemical cycle of dynein. Following ADP release from AAA1, ATP binding (step 1) causes the undocking of the linker from AAA5 and the subsequent transition from the γ registry of the stalk helices with intermediate weak MT-affinity (red) to the weakly MT-binding β registry (blue) only if AAA4 is bound to ATP or in the post-hydrolysis state (step 2). After the detachment of the rear head (step 3), which occurs when AAA3 is in the ADP ∙ Pi transition state or bound to ADP, the ‘recocking’ of the linker (priming stroke) displaces the detached head forward to a new front MT-binding site while the MT-attached head bears the load (step 4). Following ATP hydrolysis and Pi release from AAA1 (step 4), rebinding to the MT in the weakly MT-binding β registry (step 5) causes the transition from the ‘high-energy ADP* state’ to the ‘low-energy ADP state’, which generates a linker swing (powerstroke), resulting in the docking of the linker to AAA5 and the transition into the strong MT-binding α registry (yellow) of the stalk helices (step 6). A prerequisite for the strong binding state is that AAA3 is not bound to ADP, suggesting that AAA3 is still in the ADP ∙ Pi state or nucleotide free. The MT minus-end-directed linker swing generates the forward movement of dynein’s center of mass and the attached load.

In conclusion, our work demonstrates that blocking AAA4-ATP binding locks the dynein linker element in the post-powerstroke state and locks the stalk helices in the γ registry. Our data further suggests that ATP binding to AAA4 is necessary for the trailing head to undock the linker from AAA4/AAA5, which allows for the transition into the weak MT-binding β registration^24^ when AAA1 is bound to ATP and AAA3 is in the post-hydrolysis state^42^. To account for these findings, we have refined the dynein model we have recently described^24^ (Fig. 7, step 1 and step 2). After detachment from the MT (Fig. 7, step 3), the trailing head is displaced forward by the priming stroke of its linker (Fig. 7, step 4). The priming stroke can only occur when AAA4 is bound to ATP (or if it assumes the ADP.Pi transition state) to open the gate on AAA3 and when ATP is bound to AAA1. In addition, AAA3 must be in the ADP.Pi transition state or bound to ADP to open the gate on AAA1^42,56^. Following ATP hydrolysis, the tethered head then binds to a new binding site on the MT (Fig. 7, step 5). MT binding via initial weak interactions causes the power stroke (Fig. 7, step 6). The docking of the linker N-terminus to AAA5 in the post-powerstroke state then allows the transition into the strong MT-binding α-registration, which is capable of bearing sufficient force.

In summary, our data suggest that dynein uses a “two-doored”, double “key-lock” mechanism^66^ where the AAA4 and AAA3^42^ nucleotide states control the effects of AAA1-ATP binding and hydrolysis on both dynein-MT binding and linker conformational changes. Multi-layered regulation by dynein’s AAA+ domains underscores the complex interactions among AAA1, 3, and 4, dynein MTBDs, and external and intramolecular tension that collectively control dynein mechanochemistry. The results presented here will enable future studies on the role of AAA4 in dynein mechanochemistry. It is possible that AAA4 presents a locus for dynein regulation. For example, dynein’s regulatory protein lissencephaly-1 (Lis1), which is a dimer, binds to two distinct sites on dynein’s AAA+ ATPase ring, a site that overlaps with AAA3 and AAA4 (“site_ring_”) and at the base of the coiled-coil stalk (“site_stalk_”)^57,67^. It was shown that the interaction of Lis1 with site_ring_ is responsible for an increased MT affinity and reduced velocity^57,68^, while the concurrent binding to both sites induces weak MT-binding and increased velocity^57^. It is therefore possible that the reduction in dynein velocity as a result of the interaction of Lis1 with site_ring_ is due to control of AAA4 and AAA3 function (as preventing ATP hydrolysis by AAA3 or AAA4 reduces dynein velocity, Fig. 2). Future studies will be necessary to determine whether Lis1 affects dynein motion by controlling the gating functions of AAA3 and/or AAA4. The observation that the binding of Lis1 to site_stalk_ depends on the nucleotide state of AAA3 supports such a possibility^57^.

## Methods

### Generation of yeast strains

Mutant yeast stains (listed in Supplementary Table 1) were engineered as described previously^24^. Briefly, the PrimerQuest tool from IDT (Integrated DNA Technologies) was used to design PCR primers (see Supplementary Table 2 for the list of primers used), and DNA fragments were generated using KOD Hot Start DNA polymerase (EMD Millipore). Yeast transformation was performed using the Frozen-EZ Yeast Transformation II Kit (Zymo Research), followed by a two-step selection method using either synthetic media with uracil-dropout amino acid mix (SC/URA) or 5-fluorouracil (5-FOA) as the selective agents. All newly engineered and mutated yeast strains were confirmed by PCR and sequencing.

### Yeast growth and protein expression

Full-length homodimeric yeast dynein, GFP-Dyn1_471kDa_, and mutants were generated and purified as described recently^69^. Yeast cultures and protein production of the single-headed dynein, GFP-Dyn1_331 kDa_, and its mutants was done as described previously^24^ with the modifications described below. The parent strain used in this work was VY137, which expresses a tail-truncated minimal *S. cerevisiae* MD containing the linker, AAA+ ring, stalk, buttress, and MTBD. This construct retains its motor activities^42,52,56^ and is equivalent to the *Dictyostelium discoideum* MD used in key biochemical studies^19,22,37,39,70–72^. The genotype was *pGal:ZZ:Tev:GFP:HA:D6 MATa; his3-11,15; ura3-1; leu2-3,112; ade2-1; trp1-1; pep4Δ:HIS5; prb1Δ*. Mutants were expressed behind the inducible galactose promotor (*pGAL*). Yeast cells were grown in 5 mL of 2× YPD medium (20 g/L yeast extract, 40 g/L peptone, 4% [w/v] dextrose) overnight, then transferred to 50 mL of YPR solution (2% [w/v] raffinose) and inoculated for 8 h. For single-headed dynein, expression was induced by growing the cells in 2× YPG medium (4% [w/v] galactose) to a final OD_600_ between 1.5 and 2.5 (~16 h). After harvesting by centrifugation at 500 × *g* for 5 min, the pellets were resuspended in 0.2 volumes of ddH_2_O and flash-frozen in liquid nitrogen as small droplets. The cell pellets were stored at −80 °C until further use.

### Yeast dynein purification

To purify the dynein, the frozen cell pellet droplets were pulverized using a kitchen coffee grinder that had been pre-chilled with liquid nitrogen. Next, 0.25 volumes of 4× lysis buffer (1× lysis buffer: 30 mM HEPES, 50 mM KAc, 2 mM Mg(Ac)_2_, 1 mM EGTA, 10% glycerol, 1 mM DTT, 0.1 mM Mg-ATP, 0.5 mM Pefabloc, 10 ng/mL Leupeptin, 10 ng/mL Pepstatin A, and 0.2% v/v Triton X-100, pH 7.2) were added to reach a final 1× lysis buffer concentration. The cell lysate was cleared via ultracentrifugation at 80,000 × *g* for 10 min at 4 °C. Then, 0.25 mL of IgG sepharose 6 fast flow beads (GE Healthcare) were added to the supernatant and incubated for 1 h at 4 °C while rotating. The dynein-bound IgG beads were then washed with ~10 bead volumes of 1× lysis buffer, followed by a wash with 10 mL of 1× TEV protease cleavage buffer (30 mM HEPES, 150 mM KAc, 2 mM Mg(Ac)_2_, 1 mM EGTA, 10% glycerol, 0.1 mM Mg-ATP, 0.5 mM Pefabloc, and 0.1% v/v Triton X-100, pH 7.2). The beads were then resuspended in an equal volume of cleavage buffer and 2% v/v of AcTev protease (ThermoFisher Scientific) was added. The mixture was nutated at 4 °C for 2 h to cleave dynein from the IgG beads. The mixture was centrifuged at 1000×*g* for 1 min at 4 °C, and the dynein-containing supernatant was then collected, flash-frozen with liquid nitrogen, and stored at −80 °C.

### MT binding and release purification of dynein

MT binding and release purification was performed to further purify the dynein and to remove any inactive motors and aggregates. To 50 μL of TEV-released dynein, 0.1 µL of 10 mM paclitaxel and 5 μL of 5 mg/mL paclitaxel-stabilized MTs were added. This solution was then layered onto a 100 μL sucrose cushion (30 mM HEPES, 200 mM KCl, 2 mM MgCl_2_, pH 7.4, 10% v/v glycerol, 25% w/v sucrose, 1 mM DTT, and 20 μM paclitaxel) and centrifuged at 25 °C for 10 min at 50,000 × *g*. After removing the supernatant, the pellet was carefully rinsed with 60 μL of wash buffer (30 mM HEPES, 150 mM KCl, 2 mM MgCl_2_, 10% glycerol, 1 mM EGTA, 1 mM DTT, and 20 µM paclitaxel, pH 7.2) and resuspended in 52 μL of wash buffer with 6 mM Mg-ATP. After incubating for 2 min at room temperature, the solution was centrifuged for 5 min at 50,000 × *g*. The MT suspension was then collected, aliquoted in 2 μL volumes, and flash-frozen in liquid nitrogen before storing at −80 °C.

### Flow chamber preparation and MT immobilization

Flow chambers were prepared as described previously^73^. Briefly, 18 × 18 × 0.17-mm coverslips (Zeiss) were placed in a porcelain coverslip rack using forceps and submerged in HNO_3_ (25% v/v) for 15 min followed by rinsing with ddH_2_O. Then, the rack with the coverslips was placed in NaOH for 2 to 5 min, followed by rinsing extensively with ddH_2_O. After drying on a heating block for 30 min, the coverslips were store in a vacuum desiccator. The flow chambers were assembled as described^8^. To immobilize MTs on the coverslip surface, 10 μL of 5 mg/mL biotinylated α-casein was flowed into the slide chamber and incubated for 10 min. The chamber was then washed three times with 20 μL of blocking buffer (“BB”, 80 mM PIPES, 2 mM MgCl_2_, 1 mM EGTA, 1% Pluronic F-127, 1 mg/mL α-casein, and 20 μM Taxol) and incubated for 1 h to fully block the glass surface. The slide chamber was then washed twice with 20 μL of BB, and the solution inside the chamber was completely removed. Subsequently, 12 μL of 1 mg/mL streptavidin was flowed into the chamber and incubated for 10 min. The chamber was then washed three times with 20 μL of BB. Finally, 20 μL of BB that contained the suspended MTs and 10 μM paclitaxel was flowed into the chamber and immediately washed with 40 μL of BB. Polarity-marked MTs were prepared as described previously^24^.

### Single-molecule motility assay

To dimerize the single-headed dynein constructs, Cy3-labeled anti-GFP antibodies were used as previously described^24^. Dynein constructs were diluted to the appropriate concentration using HME30 (30 mM HEPES, 2 mM MgCl_2_, and 1 mM EGTA) and incubated on ice with Cy3-labeled, anti-GFP antibodies for 10 min. A final motility buffer (MB) containing 1 mM ATP, 1 mg/mL α-casein, 20 μM Taxol, 75 mM KCl, 2 mM Trolox, Gloxy, 1 mM TCEP, and the motor-antibody mixture was flowed into the chamber. MTs and dynein were visualized with a custom-built TIRF microscope equipped with an Andor iXon Ultra EMCCD. MTs were first imaged by taking a single-frame snapshot. Dynein was then imaged with an acquisition time of 500 ms and a total 500 frames were acquired for each movie. Finally, the MTs were imaged again by taking a snapshot to check for stage drift. Movies with significant drift were not analyzed. Each sample was imaged no longer than 15 min. The kymographs were generated using Fiji and the data were analyzed using a custom written MATLAB program.

### Stall-force measurements

Stall-force measurements were performed using a custom-built force-fluorescence microscope as described previously^27,50^. Briefly, TMR-labeled MTs were immobilized to glass coverslip as described above. Full-length yeast dynein was diluted to the appropriate concentrations using trapping buffer (30 mM HEPES, 2 mM MgAc_2_, 1 mM EGTA, 1 mM TCEP, 50 mM KAc, 0.75 mg/mL α-casein, 20 μM paclitaxel, 20 mM glucose, and 2 mM Trolox, pH 7.2) and incubated for 10 min on ice with anti-GFP antibody-coated ~1-μm diameter beads (980 nm, carboxyl-modified polystyrene microspheres, Bangs Laboratories). The mixture was then supplemented with 1 mM ATP and Gloxy (a glucose oxidase and catalase-based oxygen scavenging system), and flown into the slide chamber. Trapping assays were performed at 25 °C as previously described at trap stiffnesses of 0.03–0.06 pN/nm^59^.

### MT-gliding assay

The flow chamber was assembled as described above. To immobilize dynein on the coverslip surface, 10 μL of 0.4 mg/ml rabbit monoclonal anti-GFP antibody was flown into the slide chamber and incubated for 10 min. The chamber was then washed twice with 20 μL blocking buffer (“BB”, 80 mM PIPES, 2 mM MgCl2, 1 mM EGTA, 1% Pluronic F-127, 1 mg/mL α-casein) and incubated for 10 min to block the glass surface. The slide chamber was then washed twice with motility buffer (“MB”, 30 mM HEPES, 2 mM MgCl_2_, 1 mM EGTA, 1 mg/mL α-casein, 50 mM KCl, 1 mM TCEP) before 10 μL dynein solution (diluted to an appropriate concentration in MB) was flown into the slide chamber and incubated for 2 min. The flow chamber was then washed twice with 20 μL MB to remove excess dynein. A final 10 μL motility buffer containing TMR-labeled MTs and suppled with 1 mM ATP, 20 μM Taxol, 2 mM Trolox and Gloxy was flown into the chamber. Microtubules were visualized with a custom-built TIRF microscope described above and imaged with an acquisition time of 250 ms for a total 1200 frames per movie. The kymographs were generated using Fiji.

### Unbinding-force assay

Unbinding-force measurements were performed as described previously^24,42^. Briefly, polarity-marked MTs were attached to the flow chamber coverslip as described above. Yeast dynein was diluted to the appropriate concentration using trapping buffer (30 mM HEPES, 2 mM MgAc_2_, 1 mM EGTA, 20 μM paclitaxel, 20 mM glucose, and 2 mM Trolox, pH 7.2) and incubated on ice with anti-GFP antibody Fab fragment-coated ~1-μm diameter beads (980 nm, carboxyl-modified polystyrene microspheres, Bangs Laboratories) for 10 min. To remove free unbound motors, the beads were centrifuged at 4 °C for 2 min at 3000×*g*, followed by supernatant removal. The beads were then resuspended with 40 μL trapping buffer containing 0.75 mg/mL α-casein, Gloxy (a glucose oxidase and catalase-based oxygen scavenging system) and 6.6 units/mL Apyrase (to remove residual ATP for the apo state experiments) or 1 mM ATP. A bead bearing a dynein MD was then held in an optical trap as the nano-positioning stage swept back and forth parallel to a surface-bound MT. The stage speed was adjusted to produce an apparent loading rate of 5.6 pN/s (the true loading rate depends on the compliance of the motor and is smaller than the apparent rate^24^).

### Analysis of data generated by the constant-pulling assay

As we showed previously^42^, the largest forces in our constant-pulling unbinding experiments (Fig. 3a) usually occur when a bead rebinds to the MT before returning to the trap center. We call these secondary binding/unbinding events. For primary events, zero force was applied to the MD immediately after MT binding (*F*_start_ = 0), but for secondary events, *F*_start_ > 0. Because the history of force applied to the bond depends on *F*_start_, we only focused on primary events (Fig. 3a, right). Measurements from multiple beads and experiments under the same conditions were pooled together and used to generate unbinding-force histograms with 1-pN bins (as our force detection limit is ~0.3 pN, we only plotted the force-dependent unbinding rates for forces above 0.5 pN). To compare the unbinding-force distributions and the derived force-dependent unbinding rates for both loading directions, we plotted the data as a function of the absolute force values. Normalized histograms that approximated the probability density functions for unbinding at a given force were then calculated. The value of each bin was divided by *N*, the total number of unbinding-force measurements. Because the unbinding-force distributions were not normally distributed, we estimated the sampling error by bootstrapping. For each histogram, 95% confidence intervals (CIs) for the mean statistic were calculated using the MATLAB bootci() function as described previously^42^. To estimate *p*-values when comparing means of different distributions, we first created a dataset representing the sampling distribution of the mean for each original dataset. First, we bootstrapped 10^5^ means with the MATLAB function bootstrp(). We then subtracted these means pairwise to create a dataset representing the sampling distribution of the difference of the means. From each measurement in this dataset, we subtracted the mean difference of the means, which shifted the mean of the distribution to zero. This transformation was consistent with the null hypothesis of no difference between the original unbinding-force distribution means. The *p*-value (*p*_m_) was then calculated as the proportion of the bootstrapped mean differences that were greater than or equal to the difference observed between the means of the original datasets (two-tailed test). Similar to our recent work^24^, we considered the compliance of the dynein motor and bead linkage, which resulted in a force-dependent loading rate. Uncertainty arises in the calculated unbinding rates as a function of force due to limited statistics for larger forces. To reduce this uncertainty, we used a kernel density estimator to describe the probability density functions of the measured unbinding forces before transforming them into force-dependent unbinding rates^61^.

### Reporting summary

Further information on research design is available in the Nature Research Reporting Summary linked to this article.

## Supplementary information

Supplementary Information

Reporting Summary

## Data Availability

Data supporting the findings of this manuscript are available from the corresponding author upon reasonable request. A reporting summary for this Article is available as a Supplementary Information file.
