# Supplementary material for: The regulatory function of the AAA4 ATPase domain of cytoplasmic dynein
Source: Nat Commun. 2020 Nov 23;11:5952. doi: 10.1038/s41467-020-19477-3 (PMC7683685; doi:10.1038/s41467-020-19477-3)
Supplement: Supplementary file 1 — Supplementary Information [file 41467_2020_19477_MOESM1_ESM.pdf]

`

“The regulatory function of the AAA4 ATPase domain of cytoplasmic dynein”

Liu et al.

Supplementary Information

Supplementary Tables

Construct	Description	Genotype	Source
VY137	WT $Dyn1_{331kDa}$	*pGAL-ZZ-TEV-GFP-3xHA-331DYN1*	Vale lab
VY696	AAA3 E/Q - $Dyn1_{331kDa}$	*pGAL-ZZ-TEV-GFP-3xHA-331DYN1 (E2488Q)*	Vale lab
VY697	AAA4 E/Q - $Dyn1_{331kDa}$	*pGAL-ZZ-TEV-GFP-3xHA-331DYN1 (E2819Q)*	Vale lab
VY861	AAA3 K/A - $Dyn1_{331kDa}$	*pGAL-ZZ-TEV-GFP-3xHA-331DYN1 (K2424A)*	Vale lab
VY862	AAA4 K/A - $Dyn1_{331kDa}$	*pGAL-ZZ-TEV-GFP-3xHA-331DYN1 (K2766A)*	Vale lab
GY302	AAA1 E/Q, AAA3 E/Q, AAA4 E/Q - $Dyn1_{331kDa}$	*pGAL-ZZ-TEV-GFP-3xHA-331DYN1 (E1849Q, E2488Q, E2819Q)*	This study
GY304	AAA1 E/Q, AAA3 E/Q, AAA4 K/A - $Dyn1_{331kDa}$	*pGAL-ZZ-TEV-GFP-3xHA-331DYN1 (E1849Q, E2488Q, K2766A)*	This study
GY306	AAA3 E/Q, AAA4 E/Q - $Dyn1_{331kDa}$ + C-term GFP	*pGAL-ZZ-TEV-3xHA-331DYN1 (E2488Q, E2819Q)-GFP*	This study
GY308	AAA4 E/Q - $Dyn1_{331kDa}$ + C-term GFP	*pGAL-ZZ-TEV-3xHA-331DYN1 (E2819Q)-GFP*	This study
GY310	AAA1 E/Q, AAA3 E/Q, AAA4 E/Q - $Dyn1_{331kDa}$ + C-term GFP	*pGAL-ZZ-TEV-3xHA-331DYN1 (E1849Q, E2488Q, E2819Q)-GFP*	This study
GY312	AAA1 E/Q, AAA4 E/Q - $Dyn1_{331kDa}$	*pGAL-ZZ-TEV-GFP-3xHA-331DYN1 (E1849Q, E2819Q)*	This study
GY314	AAA1 E/Q, AAA4 K/A - $Dyn1_{331kDa}$	*pGAL-ZZ-TEV-GFP-3xHA-331DYN1 (E1849Q, K2766A)*	This study
GY318	AAA1 E/Q, AAA3 K/A, AAA4 E/Q - $Dyn1_{331kDa}$	*pGAL-ZZ-TEV-GFP-3xHA-331DYN1 (E1849Q, K2424A, E2819Q)*	This study
GY322	AAA4 K/A - $Dyn1_{331kDa}$ + C-term GFP	*pGAL-ZZ-TEV-3xHA-331DYN1 (K2766A)-GFP*	This study
GY326	AAA1 E/Q, AAA3 K/A, AAA4 K/A - $Dyn1_{331kDa}$	*pGAL-ZZ-TEV-GFP-3xHA-331DYN1 (E1849Q, K2424A, K2766A)*	This study
GY328	AAA3 E/Q, AAA4 E/Q - $Dyn1_{331kDa}$	*pGAL-ZZ-TEV-GFP-3xHA-331DYN1 (E2488Q, E2819Q)*	This study
GY330	AAA4 K/A – Full-length dynein	*ZZ-TEV-GFP-GS-HALOTAG-GS-3xHA-DYN1 PAC11-13xMYC::TRP nip100Δ::NAT (K2766A)*	This study

GY332	AAA3 K/A - Full-length dynein	*ZZ-TEV-GFP-GS-HALOTAG-GS-3xHA-DYN1 PAC11-13xMYC::TRP nip100Δ::NAT (K24624A)*	This study
GY336	AAA1 E/Q, AAA4 K/A - $Dyn1_{331kDa}$ + C-term GFP	*pGAL-ZZ-TEV-3xHA-331DYN1 (E1849Q, K2766A)-GFP*	This study
GY338	AAA4 K/A, AAA5 mutation - $Dyn1_{331kDa}$ + C-term GFP	*pGAL-ZZ-TEV-3xHA-331DYN1 (K2766A, K3438E, R3445E, F3446D)-GFP*	This study
GY340	AAA4 E/Q - Full-length dynein	*ZZ-TEV-GFP-GS-HALOTAG-GS-3xHA-DYN1 PAC11-13xMYC::TRP nip100Δ::NAT (E2819Q)*	This study
GY342	AAA1 K/A, AAA4 K/A - $Dyn1_{331kDa}$	*pGAL-ZZ-TEV-GFP-3xHA-331DYN1 (K1802A, K2766A)*	This study
GY344	AAA3 E/Q - Full-length dynein	*ZZ-TEV-GFP-GS-HALOTAG-GS-3xHA-DYN1 PAC11-13xMYC::TRP nip100Δ::NAT (E2488Q)*	This study
GY346	AAA1 K/A, AAA4 E/Q - $Dyn1_{331kDa}$	*pGAL-ZZ-TEV-GFP-3xHA-331DYN1 (K1802A, E2819Q)*	This study

Table S1: Yeast strains used in this work. "331DYN1" encodes amino acids 1219-4092 of Dyn1, with predicted molecular weight of 331 kDa, and single-headed dynein (see Reck-Peterson et al. Cell 2016, and remark in footnote on page 8, section "Yeast culture and dynein purification" in Nicholas et al. PNAS 2015), and WT ("wild type") represents the tail-truncated, single-headed dynein construct without AAA mutation. All yeast strains are based on W303 (*MATa/MATα {leu2-3,112 trp1-1 can1-100 ura3-1 ade2-1 his3-11,15} [phi+]*) plus *pep4Δ::HIS3, prb1Δ*.

URA primers	
u1F	gtgattctgggtagaagatcgg
u2R	gagcaatgaacccaataacgaaatc
u3F	cttgacgttcgttcgactgatgagc
u4R	cgatgatgtagtttctggtttttaa
**Primers for AAA1 E/Q**	
F	GAGTGGCTACCTCTTGCAATATAA
R	CCAAAGGGCTGCAATTTCTT
Stitch-F	TTAAAAACCAGAAACTACATCATCGCCAGGTTGGTAAGTCCCATATT
R-stitch	CCGATCTTCTACCCAGAATCACACCTACTAGAAGCCTGGAAAGA
Mutant F	GTTTCGATCAATTCAATAGGCTCGA
Mutant R	TCGAGCCTATTGAATTGATCGAAAC
**Primers for AAA3 K/A**	
F	TTAGAAACCGCGGTGCAG
R	GATTGCAGGCACCAACAATATG
Stitch-F	TTAAAAACCAGAAACTACATCATCGCGAAAGACACGACCACAGAA
R-stitch	CCGATCTTCTACCCAGAATCACAACTCAACTTATCGTTCGCAATAAC
Mutant F	ACCTGGATCTGGTGCTACAATGA
Mutant R	TCATTGTAGCACCAGATCCAGGT
**Primers for AAA3 E/Q**	
F	TCCGAAATTCCCTCTGTTTCTT
R	TGCCTTGGACCAGTGTTAAT
Stitch-F	TTAAAAACCAGAAACTACATCATCGCACGACATGCTGCTATTCTTTAC
R-stitch	CCGATCTTCTACCCAGAATCACTATGTTCTGTGGTCGTGTCTTT
Mutant F	TGTGACCAAATTAATTTACCAAAACTCGA
Mutant R	TCGAGTTTTGGTAAATTAATTTGGTCACA
**Primers for AAA4 K/A**	
F	GGTCCAAGGCAAACACTAAGA
R	TCTCCAACAAACCAGTCATACA
Stitch-F	TTAAAAACCAGAAACTACATCATCGCCCAAGATCCACCGTCATT
R-stitch	CCGATCTTCTACCCAGAATCACCCAGCTCCTCATCACAGAATG

Mutant F	CAGAACAGGTGCTACTATTTTAACTAG
Mutant R	CTAGTTAAAATAGTAGCACCTGTTCTG
**Primers for AAA4 E/Q**	
F	GTGATGAGGAGCTGGAAGTG
R	TTGGCCACTTGGGACATT
Stitch-F	TTAAAAACCAGAAACTACATCATCGGGAGAGGAGTATGATAAG CTCTTG
R-stitch	CCGATCTTCTACCCAGAATCACCAAAGTCGCTAAGATTGGAAT GAC
Mutant F	CTTGTTTAATCATTGATCAATCCAAC
Mutant R	GTTGGATTGATCAATGATTAAACAAG
**Primers for AAA5 mutation**	
F	AGAAAGGTGGCTGAACACTAC
R	GGCCTCTTTCTTCAGGTTGT
Stitch-F	TTAAAAACCAGAAACTACATCATCGTCCTCAGATCTAGGGTAAG GTTAG
R-stitch	CCGATCTTCTACCCAGAATCACGTTATCATGTGGGAGCTTGGA
Mutant F	GAAGAAGGTTTTGTTGAAAGATTAGAAAATGCAGTTGAAGATG GAAGTGTAG
Mutant R	CTACACTTCCATCTTCAACTGCATTTTCTAATCTTTCAACAAAAC CTTCTTC

Table S2: List of primers used for the generation of yeast strains. For methods of primers design and yeast transfection see Rao et al. in Methods Mol. Biol. 2018.
